# Real-Time Detection of Unauthorized Unmanned Aerial Vehicles Using SEB-YOLOv8s

**DOI:** 10.3390/s24123915

**Published:** 2024-06-17

**Authors:** Ao Fang, Song Feng, Bo Liang, Ji Jiang

**Affiliations:** 1 Yunnan Key Laboratory of Computer Technology Application, Faculty of Information Engineering and Automation, Kunming University of Science and Technology, Kunming 650500, China; 20222204183@stu.kust.edu.cn (A.F.); feng.song@kust.edu.cn (S.F.); 2 Yunnan Police College, Kunming 650223, China; 13708886871@139.com

**Keywords:** target detection, UAVs, complex backgrounds, small targets

## Abstract

Aiming at real-time detection of UAVs, small UAV targets are easily missed and difficult to detect in complex backgrounds. To maintain high detection performance while reducing memory and computational costs, this paper proposes the SEB-YOLOv8s detection method. Firstly, the YOLOv8 network structure is reconstructed using SPD-Conv to reduce the computational burden and accelerate the processing speed while retaining more shallow features of small targets. Secondly, we design the AttC2f module and replace the C2f module in the backbone of YOLOv8s with it, enhancing the model’s ability to obtain accurate information and enriching the extracted relevant information. Finally, Bi-Level Routing Attention is introduced to optimize the Neck part of the network, reducing the model’s attention to interfering information and filtering it out. The experimental results show that the mAP50 of the proposed method reaches 90.5% and the accuracy reaches 95.9%, which are improvements of 2.2% and 1.9%, respectively, compared with the original model. The mAP50-95 is improved by 2.7%, and the model’s occupied memory size only increases by 2.5 MB, effectively achieving high-accuracy real-time detection with low memory consumption.

## 1. Introduction

With the continuous innovation in social technology and the advancement of scientific and technological electronic equipment, unmanned aerial vehicle (UAV) technology has rapidly developed and been widely applied in areas such as agriculture, transportation, and the military [1,2], but this has also triggered an increase in misuse and violations, posing potential threats to social and national security [3].

Therefore, anti-drone systems are crucial for ensuring public safety, national security, and the security of critical infrastructure. However, effectively detecting drones is a prerequisite for countering them, providing essential information for anti-drone systems to assist in taking timely countermeasures.

Nowadays, due to the rapid development of deep learning target detection methods with strong feature extraction capabilities, the application of deep learning to UAV detection has gradually become a research hotspot. This approach holds advantages in terms of efficiency and cost. However, there are still some challenges in the real-time detection of UAVs by computer vision: first, UAVs are easily missed and misdetected in complex backgrounds; second, small target UAVs are frequently overlooked; and third, it is difficult to balance the detection cost and performance of the algorithms. YOLOv8 is an outstanding algorithm in the current YOLO series, having already made significant achievements in the field of computer vision. YOLOv8 adopts an anchorless detection method, which excels in terms of detection speed and accuracy. Therefore, this paper enhances YOLOv8s and proposes the SEB-YOLOv8s algorithm to balance detection performance and computational resource consumption. This is to address the challenges of detecting small target UAVs and the propensity for UAVs to be missed or misdetected in complex backgrounds. Our key contributions to this effort are outlined as follows:

• We propose the SEB-YOLOv8s algorithm for real-time detection of UAVs. Targeting the challenges of detecting small UAV targets, which are easily missed and difficult to discern in complex backgrounds, the SEB-YOLOv8s detection method significantly improves detection efficacy. This improvement comes through the integration of the SPD-Conv module, the design of the AttC2f module to maximize the use of spatial information from the feature map, and the introduction of the BRA module to balance computational costs while maintaining high detection performance.
• The design of the AttC2f module enhances the information extraction capability, which can aggregate cross-channel semantic information, capture the interactions between different dimensions, improve the use of small target information in shallow features, and improve the detection performance of small targets and complex backgrounds.
• We evaluated our proposed real-time UAV detection algorithm using the public dataset Anti-UAV. The experimental results demonstrate that it can achieve high-accuracy detection in real-time with reduced cost consumption and significantly outperforms YOLOv8s in terms of performance. The detection performance is comparable to one-fifth of YOLOv8x, while the model size remains only one-fifth of it.

This paper is structured as follows: Section 2 presents the related work. Section 3 describes the proposed method in detail. Section 4 presents the related experiments and discusses the results. Finally, Section 5 summarizes the paper and suggests future research directions.

## 2. Related Work

Existing UAV detection research not only relies on computer vision techniques but also uses other detection techniques to detect UAVs. The following are some typical research efforts.

Using deep learning neural networks with computer vision techniques to detect UAVs is a powerful approach. Previously, Mahdavi et al. [4] used deep learning neural networks with traditional machine learning methods such as SVM and KNN classification for UAV detection, respectively, and found that the neural network classifiers were more accurate. To detect UAVs at longer distances, Magoulianitis et al. [5] used super-resolution technology to enlarge the data by two times before reaching the detection system, increasing the presence of UAVs on the image screen. This, combined with the Faster R-CNN [6] detection system, improved the recall rate of the detection process. Zeng et al. [7] addressed the problem of large disparities in the sizes of UAVs by proposing a UAV detection network based on RetinaNet. Using Res2net as the backbone network, they extracted UAV target features from multiple receptive fields and designed a convolutional neural network through a novel hybrid feature pyramid structure. This structure achieves layered multi-scale feature fusion, enhancing the robustness of detection across different UAV sizes. In response to issues of missed detections, low accuracy, and slow detection speeds, Hamid R. Alsanad et al. [8] made improvements based on YOLOv3 [9] to increase the detection scale and reduce the missed detection of small UAV detection. This showed the potential to outperform traditional detection methods, but the UAV data scene with a single large target still lacks detection capabilities for small UAVs and UAVs in complex scenarios. To detect UAVs that are prone to missed detections and misdetections, Cheng et al. [10] propose the UAV detection method YOLOv4-MCA. This method selects MobileViT as the backbone network, whose lightweight feature can reduce computational costs while effectively extracting both global and local features of the UAV target. It also adopts coordinate attention to improve the path aggregation network (PANet) and optimize the anchor frame of the UAV target. This approach enhances the detection efficiency, reduces missed detections, and minimizes misdetections of UAVs. Hansen Liu et al. [11] designed real-time detection algorithms tailored for fast-moving UAVs by pruning the convolutional channel and shortcut layer of YOLOv4 [12], which improves the speed of UAV target detection but at the cost of reduced detection accuracy. Ulzhalgas Seidaliyeva et al. [13] designed real-time detection algorithms tailored for the characteristics of fast-moving UAVs to improve detection accuracy. They used a fixed camera to collect data and divided the task of detecting UAVs into two separate tasks. To improve the accuracy of UAV detection, a fixed camera was used to collect data, and the task of UAV detection was divided into two separate tasks: detecting moving objects and classifying detected objects such as UAVs, birds, and backgrounds. The detection of moving objects was based on background subtraction, while classification was carried out using a convolutional neural network (CNN). This proposed method can achieve high accuracy and processing speed in detecting UAVs, but it is highly dependent on static backgrounds and has limited adaptability to complex backgrounds. Yaowen Lv et al. [14] aimed to make full use of high-resolution UAV images to improve the accuracy of UAV detection. The authors used high-resolution images acquired by stationary cameras to detect UAVs, and proposed a detection method combining background differencing with improved YOLOv5s, which excludes background information and improves detection efficiency, but it is overly dependent on static backgrounds and is ineffective for UAV detection in complex environments. Despite these improvements, the detection of UAVs still faces challenges, as small UAV targets are difficult to detect and are easily interfered with in complex backgrounds.

In addition to image processing techniques, other sensing techniques have been applied to drone detection. Sara et al. [15] used deep learning techniques such as convolutional neural networks (CNNs), recurrent neural networks (RNNs), and convolutional recurrent neural networks (CRNNs) to detect and identify drones through the distinctive acoustic fingerprints of drones in flight. It is capable of detecting the presence of drones and identifying the type of drones, but it cannot determine accurate information about the location of the drones. M. Yaacoub et al. [16] proposed a UAV acoustic recognition method based on convolutional neural networks (CNNs) and migration learning to improve the acoustic detection capability of the anti-UAV system. The method pre-trained the CNN on a large audio dataset, AudioSet, and fine-tuned it on a custom acoustic dataset to achieve efficient classification and detection of drone sounds based on log-Meier spectral features, laying the foundation for research on sound detectors based on deep learning techniques. However, the study focuses mainly on sound detection and does not address the accurate determination of UAV positions. Existing drone detection techniques rely on deep learning, have high resource requirements, and are not easily applicable to embedded devices. Brighente et al. [17] developed the anti-drone audio surveillance sentry (ADASS), the first noise-based drone detection system that can be implemented in IoT devices. The system uses an embedded machine learning model and a compressed convolutional neural network to classify audio signals from onboard microphones, enabling it to remotely monitor flying drones. However, its effectiveness in complex noise environments needs to be further investigated. Przemysław Flak et al. [18] proposed an RF sensor grid-based UAV surveillance system using distributed sensor grids and a custom neural network architecture, which could be divided into three phases, including signal acquisition and hardware-accelerated time-frequency domain transform computation in a software-defined radio (SDR) device, an embedded computer for UAV presence detection, and UAV identification at the data fusion center. The unique advantage of the RF approach is that it enables early intrusion detection (identification of the drone’s launch sequence and indication of the operator’s position before take-off) and classification of the drone. The system not only demonstrates excellent performance in noisy simulated environments and validation in outdoor scenarios but also achieves a high degree of accuracy within the sensor network. However, it suffers from a high data transmission load and a lack of complexity in the test scenarios.

These non-image-based detection methods show good capabilities in detecting UAVs, especially in specific scenes or special situations (e.g., when audio or RF signals are strong). However, the application scenarios of non-image-based methods are often limited. The SEB-YOLOv8s algorithm proposed in this paper has broader applicability compared to existing image-based and non-image-based UAV detection methods. It performs well in detecting small target UAVs and UAVs in complex backgrounds and is also cost-effective in terms of resource usage. We believe this work will be an effective addition to the field of UAV detection. The approach is described in detail in the next section.

## 3. Methods

In this section, first, the YOLOv8 algorithm is introduced; then, the SEB-YOLOv8s network proposed in this paper for UAV target detection is described in detail to solve the problems of easily missed detection of small targets when detecting UAVs in real time and difficult detection of UAV targets in complex backgrounds.

**Figure 1:**
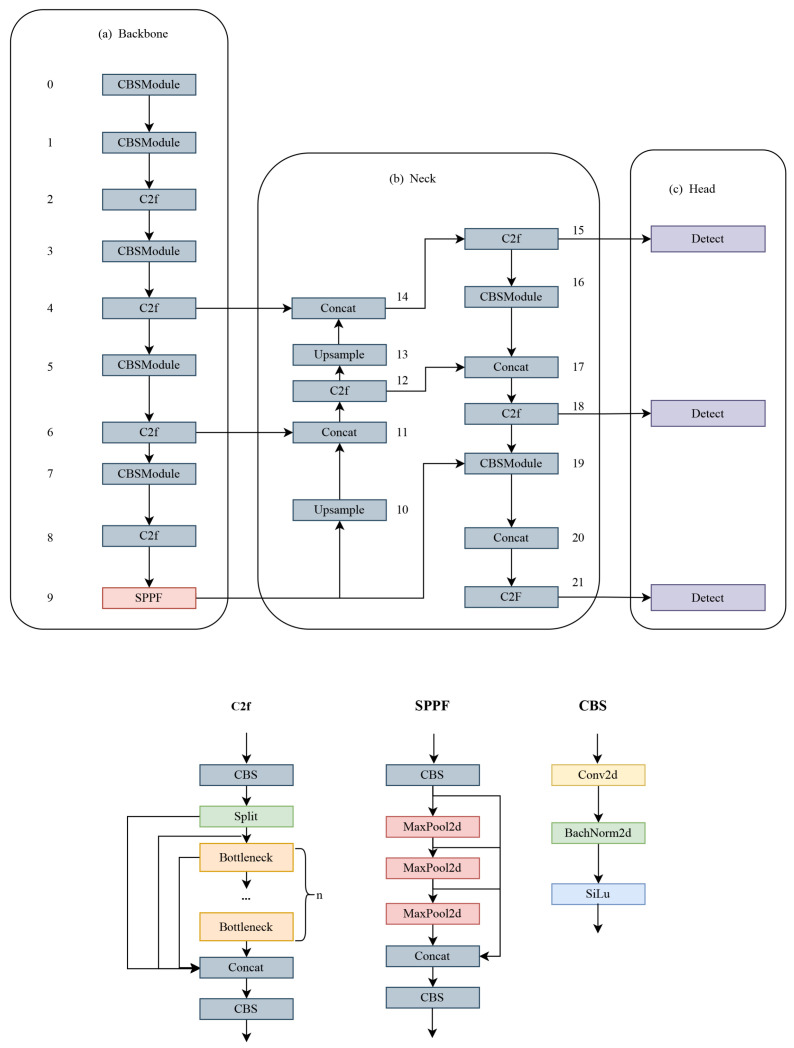
Network structure of YOLOv8.

There are five different models of YOLOv8—YOLOv8n, YOLOv8s, YOLOv8m, YOLOv8l—and YOLOv8x, and as the model size increases, the detection accuracy increases. The network structure of the model consists of three main components: the backbone, neck, and head. The network structure is shown in Figure 1, the numbers in the figure indicate the layers of the model.

In the backbone part, YOLOv8 uses the modified CSPDarknet53 as the backbone network and obtains features at different scales through the C2f module. Here, the C2f module uses a gradient shunt connection, and the cross-stage partial module (CSP) is used to perform the convolution operation with batch normalization and the SiLU activation function, and finally the feature map is output through the fast spatial pyramid pooling (SPFF) module.

In the neck part, YOLOv8 is inspired by the PANet [19] structure. Compared with the previous model, YOLOv8 simplifies the convolution operation after upsampling in the PAN structure, which reduces the computation to reduce complexity while guaranteeing performance. By combining the advantages of PAN and FPN to form a PAN-FPN top-down and bottom-up fusion feature, the shallow and deep types of information are fused to increase the information of the feature, and the quality of the feature map is improved to make it more complete and rich.

In the head part, YOLOv8 detects the head using the decoupled head structure. The structure is designed as two independent branches for bounding box regression prediction and target classification, and two different loss functions are selected separately, namely, distributed focus loss (DFL) [20] for bounding box regression prediction and the completely intersected and Complete IoU (CIoU) [21] for classification selection. This decoupled detection structure can better adapt to the characteristics of different tasks and improve the performance of the model in object classification and bounding box regression.

### 3.1. Architecture of SEB-YOLOv8s

**Figure 2:**
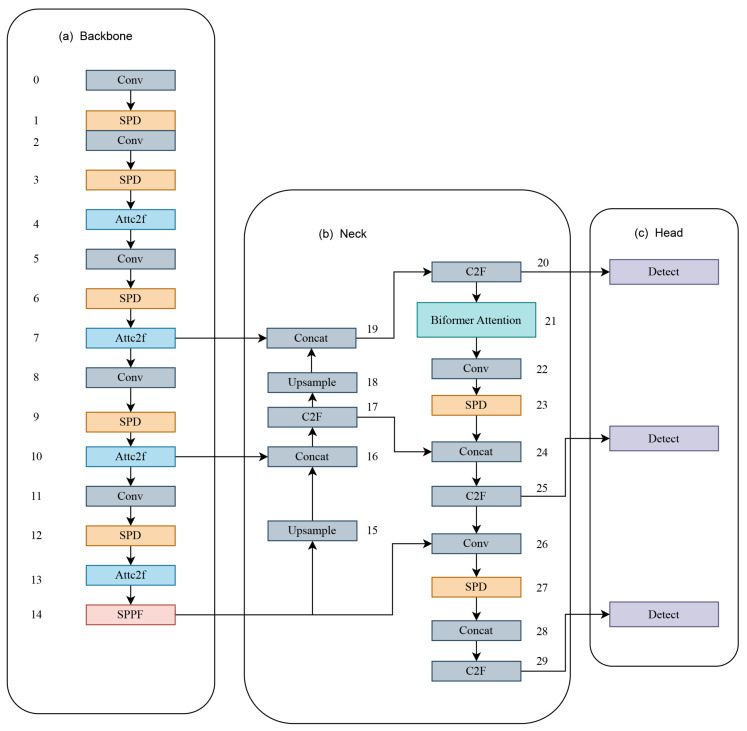
Network structure of SEB-YOLOv8s.

The framework of SEB-YOLOv8s is shown in Figure 2, the numbers in the figure indicate the layers of the model. Due to the challenges of poor detection of UAVs with small proportions in the image and difficulty detecting UAVs in complex backgrounds, YOLOv8, despite its ability to detect UAVs of various scale sizes by combining the advantages of its neck section PAN and FPN with three detection heads, does not fully meet the requirements for real-time UAV detection in diverse scenarios, especially when detecting small targets and UAVs in complex backgrounds. In order to alleviate these issues, this paper uses YOLOv8s as the base model, aiming to balance model size and detection performance. At the same time, improvements are made in terms of overall network structure construction, enrichment of extracted feature semantic information, and the attention mechanism. The main ideas of the improvement strategy are outlined as follows:

First, the SPD-Conv module is introduced to reconfigure the network structure to retain more shallow semantic information of UAVs on small targets or low-quality images. Then, in order to improve the attention to UAVs in shallow feature maps and inhibit the interference of complex backgrounds, the AttC2f module is proposed to make the model more fully utilize the spatial information of the feature maps, where the introduced EMA module has the advantage of not requiring the downscaling of the feature maps, and is able to provide a more comprehensive feature map with the introduction of an EMA module to provide high-quality pixel-level deep feature maps without the need to downscale the feature maps. Finally, a two-layer routing attention mechanism is introduced to filter out the most irrelevant regions by screening the feature maps to increase attention to the UAV target with less computation, which can take into account both the real-time detection speed and better detection performance.

#### 3.1.1. Enhanced Feature Detail

When detecting UAVs, their proportion in the image is relatively small, making it difficult to detect small UAV targets at long range. Although deep learning neural networks have made significant contributions across various fields, the step convolution and pooling operations used for extracting small target features still incur losses, especially in images with low pixel counts or small targets. UAVs are categorized into large, medium, and small sizes, and while YOLOv8’s multi-scale detection facilitates the detection of UAVs, the multi-scale fusion typically involves stridden convolution and maximum pooling. As the network depth increases, problems such as loss of detailed information and insufficiently accurate feature representation arise. Traditional convolution can learn limited features; thus, for the extraction of small target features or unclear images, the results are often unsatisfactory. In this paper, we introduce a non-strided convolution or pooling module (SPD-Conv) [22] to readjust the network structure.

SPD-Conv consists of two parts: a space-to-depth (SPD) layer and a non-strided convolution. First, the SPD part applies the original image transformation technique [23] extension within the neural network to downsample the feature maps and then undergoes a non-strided convolution (stride = 1) to further transform the feature maps. SPD downsamples the original feature map *X* by slicing it into multiple sub-feature maps and connecting them along the channel dimension to form $X^{\prime }$. Each sub-feature map is a subset of the original feature map with some degree of downsampling. After the SPD feature transformation layer, the downsampled feature maps $X^{\prime }$ are further transformed into $X^{\prime }$ by adding a non-strided convolutional layer to reduce the channel dimension and extract more discriminative feature information. This approach helps to reduce the computational burden and accelerate the processing speed of the network while preserving important information. In this paper, we replace the strided convolution in the CBS module of the original model with non-strided convolution, using SPD in conjunction with non-strided convolution to adjust the network structure, minimizing the feature loss of UAV targets and retaining more shallow semantic information.

#### 3.1.2. Replacing C2f with AttC2f Module

**Figure 3:**
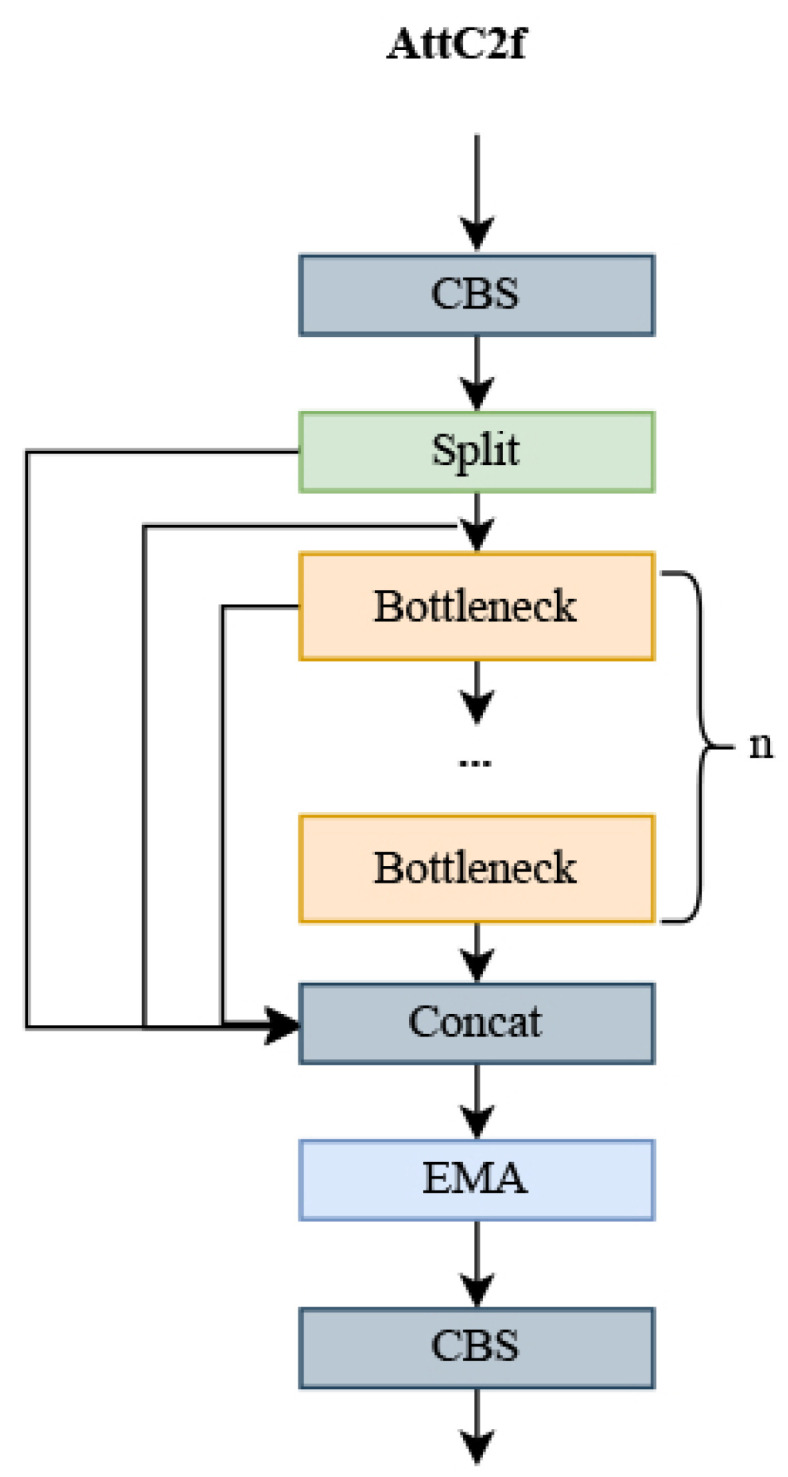
AttC2f module.

For UAV detection in complex backgrounds, where the details or features of the background and the UAV intersect and the UAV target is a small part, it is common for features to be either not extracted or misclassified as background features, resulting in detection leaks. To address this, the C2f module has been improved with the introduction of a new module called AttC2f, whose structure is shown in Figure 3. This modification enhances the focus on UAV targets with minimal impact on computation. The efficient multi-scale attention module (EMA) [24] is introduced into the AttC2f module; the EMA module employs multi-scale convolution through parallelization and aggregation of semantic information across spatial channels for learning. The selection of convolution kernels of different sizes through branching allows the CNN to collect spatial information at various scales within the same feature extraction phase. Additionally, the introduction of parallel subnetwork blocks aids in efficiently capturing interactions between different dimensions and establishing dependencies between them, effectively capturing long-range dependencies and precise positional information, thus enhancing the pixel-level attention of the convolutional neural network (CNN) to high-level feature maps.

**Figure 4:**
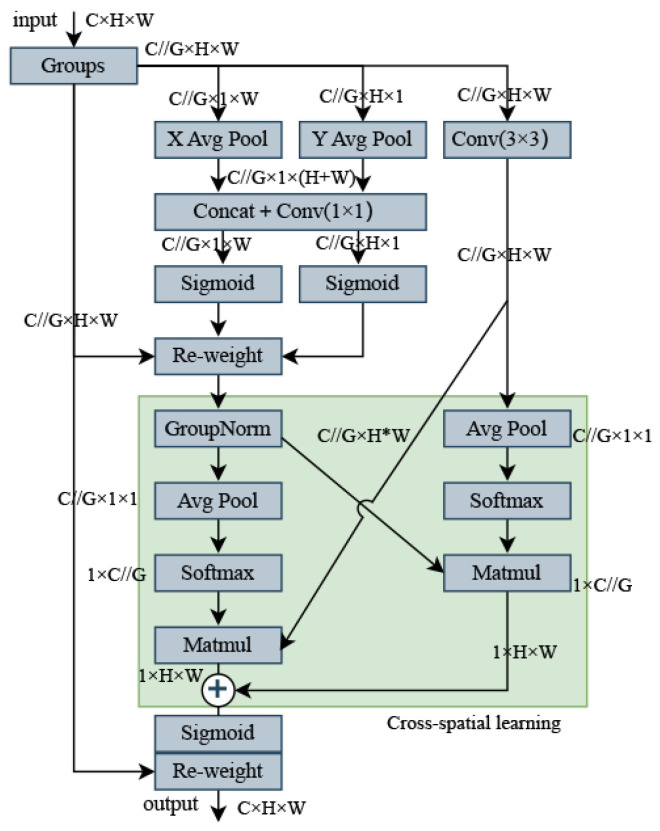
EMA module.

In contrast to channel or spatial attention mechanisms, which model cross-channel relationships by reducing the dimensionality of the channels, reducing channel dimensionality to model cross-channel relationships can have a number of implications and drawbacks. First, channel dimensionality reduction may lead to information loss, as fewer channels may not fully capture all the details of the original data. Second, the computational cost may increase, and although the 1×1 convolution is relatively lightweight, the computational overhead still needs to be considered in large networks. The choice of hyperparameters also becomes a challenge, and inappropriate choices can lead to performance degradation. In addition, channel dimensionality reduction may not apply to all tasks, and its effectiveness may depend on the characteristics of the task and the characteristics of the data. Finally, channel dimensionality reduction may reduce the interpretability of the model, as fewer channels may not be as intuitive as the original channels. The EMA module efficiently learns channel descriptions while preserving channel dimensionality in convolutional operations, and provides better deep feature maps for generating better pixel-level attention. The overall structure of EMA is illustrated in Figure 4.

Feature grouping: The EMA module divides the input feature map *X* $\in R^{H\times W\times C}$ into multiple cross-channel dimensional sub-features *G* by channel grouping, where the groups can be represented as $X=[X_{0},X_{i},...,X_{G-1}]$, where $X_{i}\in R^{H\times W\times C}$ denotes the *i*th sub-feature, each of which is used to learn different semantic information. This helps the model to better understand the relationship between the different channels in the input feature map and allows the learned attention weights to fine-tune the feature representation of the region of interest in each sub-feature.

Parallel substructure: Different cross-channel interaction features are implemented between two parallel paths in the 1×1 branch, aggregating the two-channel attention graphs within each group by simple multiplication. And in the 3×3 branch, local cross-channel interactions are captured by 3×3 convolution to expand the feature space. Non-linearity is introduced on top of the linear convolution to better model the relationship between features, and the EMA module not only encodes the inter-channel information to adjust the importance of different channels but also retains the precise spatial structure information in the channels. This design helps to better understand and exploit the semantic information of the input feature maps.

Cross-channel learning: A method for aggregating cross-spatial information in different spatial dimension directions to achieve richer feature aggregation. Two tensors are introduced, one from the output of a 1×1 branch and the other with the output of a 3×3 branch. The authors use 2D global average pooling to encode the global spatial information in the output of the 1×1 branch, while the output of the 3×3 branch is directly converted into the corresponding dimensional form before the joint activation mechanism of the channel features. The 2D global pooling operations are designed to encode global information and model long-range dependencies, as follows:

(1) $$Z_{c}=\frac{1}{H\times W}\sum_{j}^{H}\sum_{i}^{W}x_{c}\left(i,j\right)$$

To make the computation more efficient, the authors apply the Softmax function to the output of the 2D global average pooling to fit a linear transformation. The output of this parallel processing is then multiplied by a matrix dot product operation to obtain the first spatial attention map. This map collects spatial information at different scales in the same processing stage. Similarly, the authors also employ 2D global average pooling in the 3×3 branch to encode global spatial information, while the 1×1 branch is directly transformed into the corresponding dimensional form prior to the joint activation mechanism of the channel features. The output feature maps within each group are then computed using a sigmoid function, aggregating the two spatial attention weights generated. The final output of the EMA is the same size as *X*, which is both efficient and effective for stacking in modern architecture.

Through a cross-space learning approach, the EMA module can model long-range dependencies and embed precise positional information about the UAV. Fusing contextual information at different scales enables CNN to produce better pixel-level attention for high-level feature maps. Contextual information between intermediate features is better utilized by using 3×3 and 1×1 convolutions in parallel, more efficiently modeling short-range and long-range dependencies for improved performance compared to incremental behavior with gradually formed finite sensory fields. Doing so allows the model to bring less computation while focusing more on UAV features. In this paper, to fully utilize this module, we introduce the proposed AttC2f module into the backbone network, replacing four of the C2f modules.

Using a cross-space learning approach, the EMA module can model long-range dependencies and embed precise positional information from the UAV. By fusing contextual information at different scales, the CNN can produce better pixel-level attention for high-level feature maps. Contextual information between intermediate features is better exploited by using 3×3 and 1×1 convolutions in parallel to more efficiently model short-range and long-range dependencies for improved performance compared to incremental behavior with gradually formed finite sensory fields. This allows the model to use less computation while focusing more on UAV characteristics. In this paper, we introduce the proposed AttC2f module into the backbone network, replacing four of the C2f modules, to fully exploit this module.

#### 3.1.3. Introducing Bi-Level Routing Attention Mechanism

**Figure 5:**
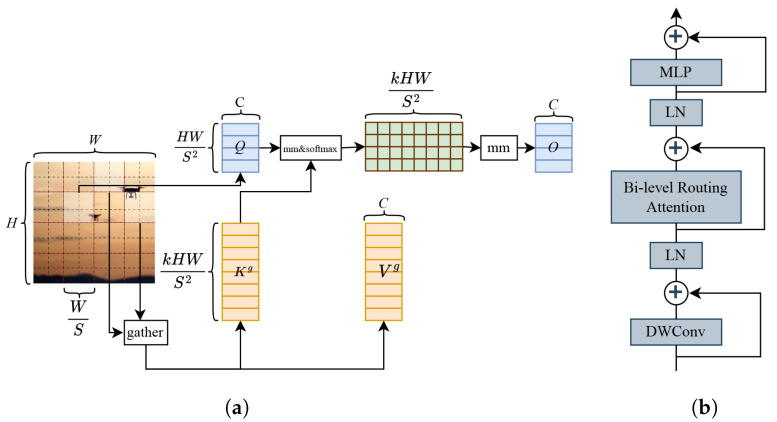
Graphical data: (**a**) bi-level routing attention steps, and (**b**) bi-level routing attention module.

In traditional attention mechanisms, each query computes attention weights with all key–value pairs, which can lead to an increase in computational complexity when dealing with long sequences. The sparse attention mechanism [25] reduces the computational overhead by restricting each query to compute attention with only a small number of key–value pairs. In contrast to conventional sparse attention mechanisms, bi-level routing attention (BRA) is a dynamic, query-aware sparse attention mechanism that eschews a static scheme and avoids sharing a sampled subset of key–value pairs among all queries. Instead, it filters out the least relevant key–value pairs at the coarse region level, thus retaining only a small fraction of the routing regions. For Transformers, the multi-head attention mechanism (MHSA) is commonly used, but it faces scalability issues due to each of the *N* queries processing *N* key–value pairs. The core idea of BRA is to introduce the notion of regions, and the Dynamic Sparse Attention Mechanism does not require each query to focus on all key–value pairs. Instead, it focuses on a small number of key–value pairs through regional-level queries and keys, as well as the indexing of routing regions, to reduce the computational complexity of the self-attention mechanism. Finally, the final output is obtained by performing an attention-weighted summation of the values, along with some shape transformations, whose workflow diagram and structure are shown in Figure 5.

The steps of the BRA workflow are as follows:

(1) Region subdivision and linear projection. The 2D feature map *X* $\in R^{H\times W\times C}$ is used as input, where *X* $\in R^{S^{2}\times \frac{HW}{s^{2}}\times C}$ is divided into non-overlapping blocks of regions S×S. The query, key, and value tensors, *Q*, *K*, *V* $\in R^{S^{2}\times \frac{HW}{s^{2}}\times C}$ are generated using a linear projection, as follows:

(2) $$Q=X^{r}W^{q},K=X^{r}W^{k},V=X^{r}W^{v}$$

(2) Region-to-region routing uses directed graphs. A directed graph is constructed to identify the involvement relationships (the regions to which each region should pay attention). First, the averages of each region are applied separately to *Q*, *K* $\in R^{S^{2}\times C}$, yielding region-level queries and keys $Q^{r}$, $K^{r}$. Then, matrix multiplication is performed between $Q^{r}$ and the transpose of $K^{r}$, yielding the adjacency matrix $A^{r}$$\in R^{S^{2}\times S^{2}}$ of the region-to-region association graph, i.e.,

(3) $$A^{r}=Q^{r}{\left(K^{r}\right)}^{T}$$

The entries in $A^{r}$ are used to measure the semantic correlation between two regions. Then, the association graph is simplified by retaining only the topk connections for each region. Specifically, the row-wise topk operator (topk index) is used to derive the way out by indexing the matrix $I^{r}\in N^{S^{2}\times k}$, i.e.,

(4) $$I^{r}=topkIndex\left(A^{r}\right)$$

Therefore, the *i*-th row of $I^{r}$ contains the indices of the topk regions most relevant to the *i*-th region.

(3) Token-to-token attention. Leveraging the routing index matrix $I^{r}$ for region-to-region routing, fine-grained token-to-token attention is implemented. This is achieved by first aggregating the key and value tensors $K^{g}$, $V^{g}$, and then introducing a local context enhancement term LCE(V) to focus the attention onto the aggregated key–value pairs. This ensures that each query token within each region focuses on all key–value pairs within the joint interior of the *k*-routed regions indicated by the indices.

(5) $$K^{g}=gather\left(K,I^{r}\right),V^{g}=gather\left(V,I^{r}\right)$$

(6) $$O=Attention\left(Q,K^{g},V^{g}\right)+LCE\left(V\right)$$

where the Gather operator is used to gather the key and value tensors from different regions based on the routing index matrix, and then the Attention operator is used to focus on the gathered key–value pairs.

In this paper, we introduce the BRA module at the neck. BRA can efficiently capture and process the direct remote associations present in the data, offering similar modeling effects compared to traditional attention mechanisms. In addition, BRA has a lower time complexity and higher computational efficiency than traditional attention mechanisms, consuming less computational cost for improved detection performance in UAV detection scenarios.

## 4. Experiments and Results

In this section, we use the publicly available Anti-UAV dataset to validate the effectiveness of the method proposed in this paper through a series of experiments. First, the dataset used is introduced, then the experimental environment and training strategy are presented, then the evaluation metrics used are introduced, the experimental results are discussed, and then the method proposed in this paper is compared with experimental results using other algorithms.

### 4.1. Presentation of Experimental Data

**Figure 6:**
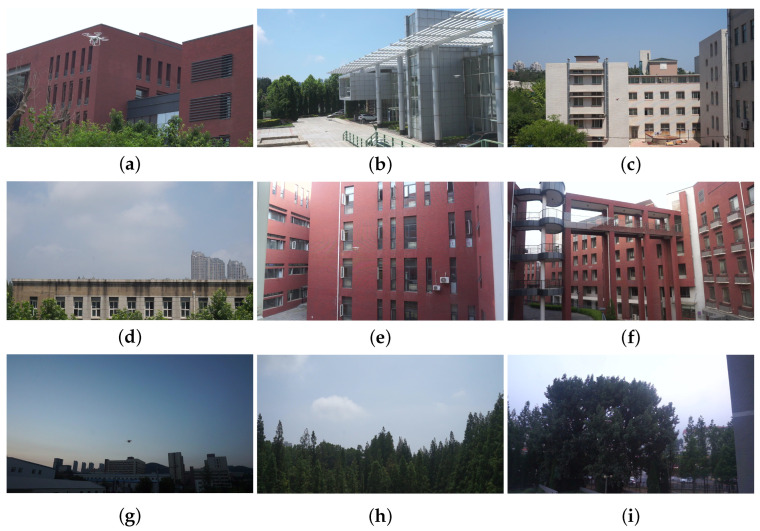
Partial presentation of the Anti-UAV dataset includes: (**a**–**c**) daytime with building background; (**d**–**f**) daytime with complex building background; (**g**) evening with open background; (**h**,**i**) outdoor with complex background.

In order to verify the effectiveness and applicability of the algorithm proposed in this paper, the publicly available dataset Anti-UAV [26] is used. This UAV dataset, collected by Dalian University of Science and Technology, contains 10,000 UAV target detection images and 20 tracking video datasets. For this paper, only the images are used. The dataset includes images taken in a variety of outdoor environments such as the sky, dark clouds, jungle, skyscrapers, residential buildings, farmland, and playgrounds. In addition, the dataset covers different lighting conditions (e.g., day, night, dawn, and dusk) and a variety of weather conditions (e.g., sunny, cloudy, and snowy). The target images contain more than 35 types of UAVs, with UAV target scales distributed between 35 pixels × 20 pixels to 110 pixels × 80 pixels, an average target area scale of about 0.013, a minimum target area scale of 1.9 × 10^−6^, and the largest target accounting for 0.7 of the whole image. All data images have been manually and accurately annotated, including target category and bounding box coordinates, etc. The annotation format is converted to the data format required for YOLOv8 model training. Applying the original dataset partitioning method, the training set comprises 5200 images, the validation set 2600 images, and the test set 2200 images. Some examples of the dataset are shown in Figure 6.

### 4.2. Experimental Environment and Training Strategies

In this paper, the experimental equipment used is as follows: server NVIDIA A40, with 45 GB of video memory (Nvidia, Santa Clara, CA, USA). The experiments were carried out under the Linux operating system, using the PyTorch framework version 2.0.1, Python version 3.9, and CUDA 11.7. YOLOv8 version 8.0.1 was used, the initial learning rate was set to 0.01, epochs were set to 300, and the batch size was 18. To reduce the computational burden on the device, we set the input data image size to 640×640. YOLOv8s was chosen as the baseline model due to the relative balance between performance and limited device resources. During model training, after several experiments for parameter debugging, the best parameter configuration was finally selected. Some important parameter settings are shown in Table 1. During the training process, CIoU was used as the bounding box loss function, and DFL (distribution focal loss) was used as the target classification loss function.

### 4.3. Evaluation Indicators

The evaluation metrics used are precision, recall, mean average precision (mAP), mAP50, mAP50-95, the number of parameters, the speed of detection (frames/milliseconds), and model size. The parameters used in the above evaluation indicators are as follows: TP (true positive) indicates that the model correctly predicts the number of samples in the positive category. FP (false positive) indicates the number of samples in the negative category that the model incorrectly predicts as being in the positive category. FN (false negative) indicates the number of samples in the positive category that the model incorrectly predicts to be in the negative category. TN (true negative) indicates that the model correctly predicts the number of samples in the negative category. Average precision (AP), for each category, measures the model’s performance in that category. Intersection over Union (IoU) measures the accuracy of a model by calculating the degree of overlap between the model’s predicted bounding box and the actual target bounding box. The meaning of the evaluation indicators and their formulas are as follows:

Precision refers to the proportion of predictions that are correct out of all outcomes where the prediction is positive.

(7) $$\mathrm{Precision}=\frac{\mathrm{TP}}{\mathrm{TP}+\mathrm{FP}}$$

Recall refers to the proportion of correct predictions among all outcomes with a true value of the positive.

(8) $$\mathrm{Recall}=\frac{\mathrm{TP}}{\mathrm{TP}+\mathrm{FN}}$$

Mean average precision (mAP) is the average of the APs of all categories. mAP combines the detection precision and recall of different categories.

(9) $$\mathrm{mAP}=\frac{1}{N}\sum_{i=1}^{N}{\mathrm{AP}}_{i}$$

### 4.4. Ablation Experiment

To prove the effectiveness of the experimental strategy, ablation experiments were performed using the publicly available UAV dataset, Anti-UAV; the experimental results are shown in Table 2. The three modules—SPD-Conv, AttC2f, and BRA—were added to the original YOLOv8s algorithm, respectively, and the precision, recall, mAP50, and mAP50-95 were obtained for the respective algorithm sets.

The experimental results in Table 2 show that the application of each enhancement strategy has improved the detection performance to different degrees. SPD can reduce the loss of detailed information in the feature map and extract more small target features, thus improving the mAP50-95 by 2.0%. The AttC2f module is designed to replace the four C2f modules in the backbone network, in which the EMA attention mechanism is introduced to enable the collection of spatial information at different scales and effective learning of channel descriptions, resulting in a 1.6% improvement in accuracy. In the neck of the network, the BRA module is introduced, which focuses on the key information in the feature map after filtering out the least relevant regions, resulting in a 1.5% improvement in mAP50-95, which is highly efficient while improving detection performance.

The model is improved to make better use of shallow features, to reduce the leakage detection rate of small target UAVs, and to improve detection performance in complex backgrounds. And each enhancement strategy has different degrees of detection performance improvement, further validating the feasibility of each enhancement scheme.

### 4.5. Comparison Experiment

In order to prove the effectiveness of the improved model, the improved model is compared with the original model in a comparison experiment while keeping other training conditions the same, and the experimental results are shown in Table 3. From the experimental results, it can be seen that the value of mAP50-95 of the improved model is increased by 2.7%, the mAP50 is increased by 2.2%, the precision is increased by 1.9%, and the recall is increased by 1.3%. The SEB-YOLOv8s algorithm proposed in this paper has a better detection capability than the original YOLOv8s algorithm.

**Figure 7:**
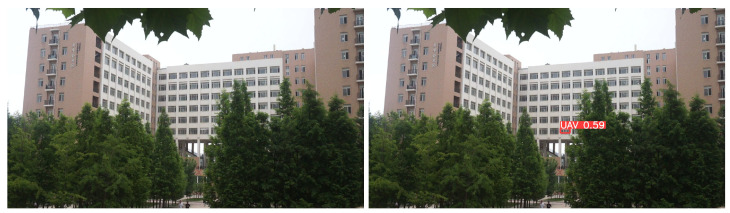

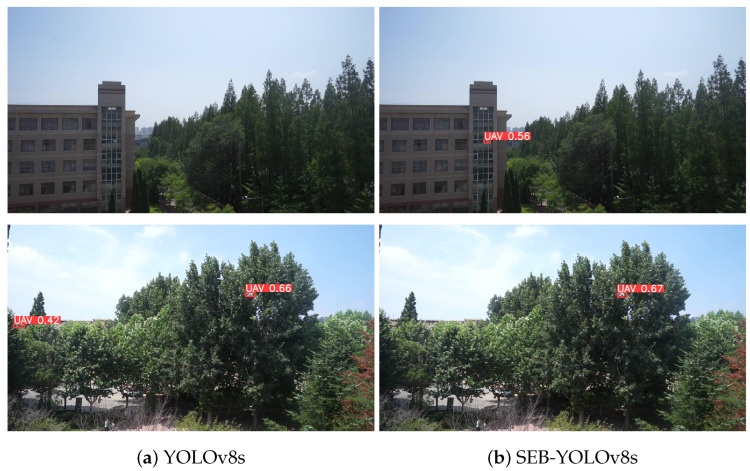
Inference results of YOLOv8s and the proposed algorithm SEB-YOLOvs on the Anti-UAV dataset. (**a**) Inference results of YOLOv8s; (**b**) Inference results of the proposed algorithm SEB-YOLOv8s in this paper.

**Figure 8:**
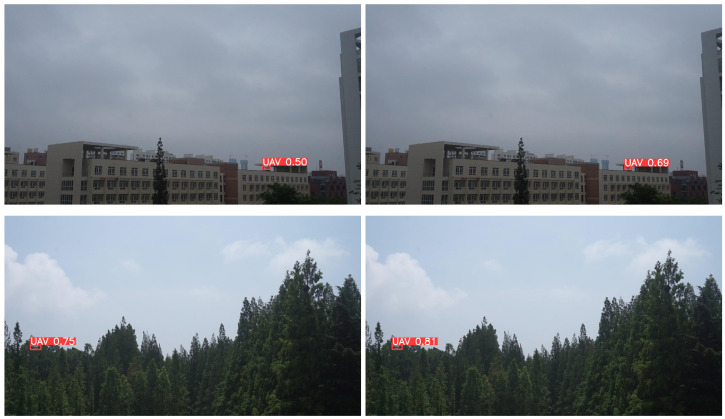

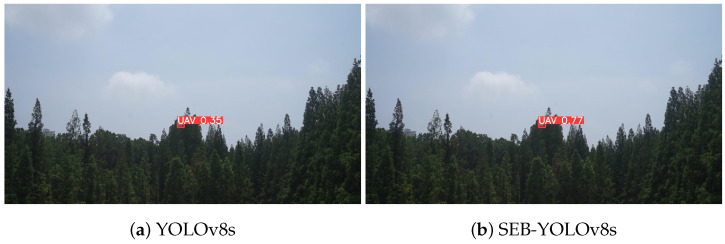
Inference results of the original model and the method proposed in this paper for UAV target detection. (**a**) Inference results of YOLOv8s; (**b**) inference results of the proposed algorithm SEB-YOLOv8s in this paper.

The detection of small UAV targets in complex backgrounds is particularly challenging, as these targets are highly susceptible to being missed when their colors blend with the environment, but the algorithm proposed in this paper effectively mitigates these problems, as shown in the reasoning results in Figure 7, in (a), the YOLOv8s algorithm experiences false detections and misses when detecting drones and small targets of drones in complex backgrounds, while in (b), the algorithm proposed in this paper accurately detects drones in complex backgrounds and can reduce the miss detection of small UAV targets. Furthermore, as shown in Figure 8, the YOLOv8s reasoning result in (a) and the reasoning result (b) of the algorithm proposed in this paper demonstrate that the confidence level for UAV detection is significantly improved with the proposed algorithm.

**Figure 9:**
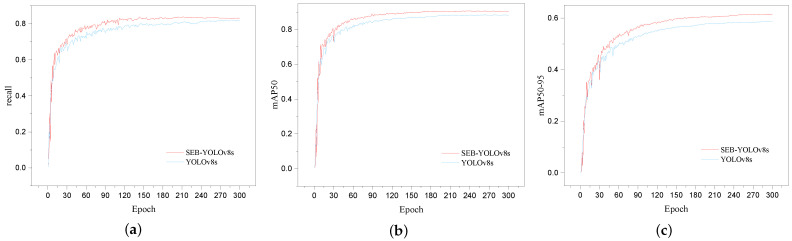
(**a**) Training curves of SEB-YOLOv8s and YOLOv8s in the recall; (**b**) training curves of SEB-YOLOv8s and YOLOv8s in mAP50; (**c**) training curves of SEB-YOLOv8s and YOLOv8s in mAP50-95.

Figure 9 shows the change curves of some important evaluation metrics of our proposed model and YOLOv8s during the training process. From Figure 9, we can see that—after 45 epochs—our proposed model achieves higher recall, mAP50, and mAP50-95 compared to YOLOv8s, indicating better performance across these three key metrics. Compared to the original model, the model proposed in this paper demonstrates better adaptability to the UAV detection task.

In order to thoroughly verify the performance of the UAV target detection model proposed in this paper, the model proposed in this paper is compared with YOLOv8n, YOLOv8s, YOLO8m, YOLO8l, and YOLO8x in comparison experiments. The experimental results are shown in Table 4. From the experimental results, it can be seen that the proposed model has the highest precision and mAP50, which are 95.9% and 90.5%, respectively, and the model size is less than a fifth of that of YOLOv8x, while the mAP50-95 and recall are only 0.5% and 1.1% lower than those of YOLOv8x. Compared with YOLOv8m, the detection speed of SEB-YOLOv8s is only 0.27 ms slower, yet the model size is less than half that of YOLOv8m. The algorithm proposed in this paper is optimal in terms of balancing detection performance and model size.

According to the experimental results in Table 5, the model proposed in this paper has the best detection performance compared with other excellent target detection models, and the model sizes of the model proposed in this paper are 26.2%, 11.9%, and 13.9% higher in precision, recall, and mAP50, respectively, with the advantage of being more than three times smaller than the SSD model. Compared to YOLOv5s, the proposed model percentages are 1.6%, 1.5%, and 2.4% higher in precision, recall, and mAP50, respectively. Compared to YOLOv7tiny, although the size of the model is larger, it outperforms it in terms of detection performance by 14.7% in recall, 2.3% in precision, and 12.6% in mAP50.

In summary, the SEB-YOLOv8s model proposed in this paper has superior detection performance compared to other models. The model can better preserve the shallow features of the target, which is especially important for the detection of small UAV targets. In addition, SEB-YOLOv8s makes better use of the channel information of the feature map to provide more accurate target position information. This enables SEB-YOLOv8s to demonstrate significant advantages over other models in the comparative experiments for small target detection and detection tasks in complex backgrounds. Therefore, our experimental results outperform the other models.

## 5. Conclusions

In addressing the challenges of UAV target detection, we face the problems of small objects that occupy a small portion of the image frame and are difficult to detect under complex backgrounds. We propose an innovative approach, SEB-YOLOv8s. First, the SPD-Conv module is introduced, consisting of a space-to-depth (SPD) layer and a non-spanning stepwise convolution, which can efficiently extract features of small UAV targets, thus reducing the leakage detection rate. Second, the AttC2f module is proposed to replace the C2f module, enhancing pixel-level attention, providing better depth feature maps, and significantly improving the detection of small and UAV targets in complex backgrounds. Finally, we introduce BiFormer, an efficient dynamic sparse attention mechanism, into the backbone network. This improves the model’s focus on key information in the feature map and further optimizes detection performance. This series of innovative enhancements aims to address the current challenges of UAV target detection and improve the model’s adaptability and performance. The experimental results show that the improved YOLOv8s algorithm enhances UAV detection performance in terms of precision and mAP50 by 1.9% and 2.2%, respectively, over the original model, and mAP50-95 by 2.7% over the original model. The improved algorithm effectively reduces the misdetection rate of UAVs while maintaining detection speed and reducing the leakage phenomenon during UAV detection. Although the algorithm proposed in this paper strikes a relative balance between detection performance and model size, the size of the improved model is still increased. In future research, we will investigate and develop more efficient lightweight models and further improve the performance of real-time UAV detection by attempting to collect and use more diverse UAV data by augmenting and extending the data.

At present, the drone market is developing rapidly, and how to effectively monitor and counteract drones that are flying and being used illegally is a difficult problem today. The current lack of public UAV datasets also poses a challenge for research, and subsequent research will target UAV detection in more disturbed environments. As only a few public UAV datasets exist, the number of datasets collected by ourselves is limited, and collecting different types of UAVs in many different scenarios will consume a lot of material and human resources, which brings great difficulty to UAV target detection.

## Figures and Tables

**Table 1:** Some of the key hyperparameters during model training.

Parameters	Setup
Initial learning rate	0.01
Final learning rate	0.0001
Epochs	300
Batch size	18
Weight decay	0.005
Warm-up epochs	3.0
Warm-up momentum	0.8
Momentum	0.937
Optimizer	SGD

**Table 2:** Detection results after introducing different improvement strategies. (✓indicates that this improvement strategy has been adopted).

Baseline	SPD-Conv	AttC2f	BRA	Precision/%	Recall/%	mAP50%	mAP50-95/%
YOLOv8s				94	81.8	88.3	58.8
	✓			93.8	83.7	89.6	60.8
	✓	✓		95.4	82.4	89.9	60.8
	✓	✓	✓	95.9	83.1	90.5	61.5

**Table 3:** Results of comparison experiments with the original model.

Models	Precision/%	Recall/%	mAP50/%	mAP50-95/%
YOLOv8s	94	81.8	88.3	58.8
Ours	95.9	83.1	90.5	61.5

**Table 4:** Comparison results of the target detection model with the proposed model. (Bold data in the table indicate the best results).

Models	Precision/%	Recall/%	mAP50/%	mAP50-95/%	Detection Time/ms	Model Size/MB	Parameter/$10^{6}$
YOLOv8n	93.7	79.5	86.5	55.3	**10.3**	**6.2**	**3.0**
YOLOv8s	94	81.8	88.3	58.8	11.5	22.5	11.1
YOLOv8m	95.3	82.7	89.6	60.2	14.4	52	25.8
YOLOv8l	95.7	82.1	90.2	61	16.4	87.6	43.6
YOLOv8x	94.4	84.2	90.4	**62**	16.8	136.7	68.1
ours	**95.9**	83.1	**90.5**	61.5	14.7	25	12.3

**Table 5:** The results of comparison of target detection models. (Bold data in the table indicate the best results).

Models	Precision/%	Recall/%	mAP50/%	Model Size/MB
YOLOv5s	94.3	81.6	88.1	18.5
YOLOv7tiny	93.6	68.4	77.9	**12.3**
SSD300	69.7	71.2	76.6	90.6
Ours	**95.9**	**83.1**	**90.5**	25

## Data Availability

Data are contained within the article.
